# Tight junction *CLDN2* gene is a direct target of the vitamin D receptor

**DOI:** 10.1038/srep10642

**Published:** 2015-07-27

**Authors:** Yong-guo Zhang, Shaoping Wu, Rong Lu, David Zhou, Jingsong Zhou, Geert Carmeliet, Elaine Petrof, Erika C. Claud, Jun Sun

**Affiliations:** 1 Department of Biochemistry, Rush University, Chicago, Illinois, USA; 2 Department of Pathology, University of Rochester, Rochester, New York, USA; 3 Department of Physiology, Kansas City University of Medicine and Bioscience, Kansas City, Missouri, USA; 4 Lab of Experimental Medicine and Endocrinology, Katholieke Universiteit Leuven, Leuven, Belgium; 5 Department of Medicine, GI Diseases Research Unit and Division of Infectious Diseases, Queen’s University, Ontario, Canada; 6 Departments of Pediatrics and Medicine, The University of Chicago Medical Center, Chicago, Illinois, USA

## Abstract

The breakdown of the intestinal barrier is a common manifestation of many diseases. Recent evidence suggests that vitamin D and its receptor VDR may regulate intestinal barrier function. Claudin-2 is a tight junction protein that mediates paracellular water transport in intestinal epithelia, rendering them “leaky”. Using whole body VDR^-/-^ mice, intestinal epithelial VDR conditional knockout (VDR^ΔIEC^) mice, and cultured human intestinal epithelial cells, we demonstrate here that the *CLDN2* gene is a direct target of the transcription factor VDR. The Caudal-Related Homeobox (Cdx) protein family is a group of the transcription factor proteins which bind to DNA to regulate the expression of genes. Our data showed that VDR-enhances Claudin-2 promoter activity in a Cdx1 binding site-dependent manner. We further identify a functional vitamin D response element (VDRE) 5΄-AGATAACAAAGGTCA-3΄ in the Cdx1 site of the Claudin-2 promoter. It is a VDRE required for the regulation of Claudin-2 by vitamin D. Absence of VDR decreased Claudin-2 expression by abolishing VDR/promoter binding. *In vivo*, VDR deletion in intestinal epithelial cells led to significant decreased Claudin-2 in VDR^-/-^ and VDR^ΔIEC^ mice. The current study reveals an important and novel mechanism for VDR by regulation of epithelial barriers.

Tight junction (TJ) structural components determine epithelial polarization and intestinal barrier functions^1^,^2^,^3^,^4^. Claudins, with approximately 24 members, are integral membrane proteins and components of tight junctions^5^. The complex expression pattern of claudins creates diversity in the barrier/channel property of TJs, which varies depending on the type of epithelium^6^. Claudin-2 and -10 tend to make tight monolayers leakier^5^,^7^,^8^,^9^. Claudin-2, a “leak” protein uniquely restricted to the proliferative zone of the crypt base^10^,^11^ , forms a paracellular water channel that mediates paracellular water transport in epithelia and renders it more “leaky”^11^,^12^,^13^,^14^,^15^. Defective epithelial barrier function has been implicated in IBD^16^ and similarly, elevation of Claudin-2 is associated with active IBD^12^,^17^. In addition, Claudin-2 likely participates in cellular functions other than its known effects on TJ function. For example, we have demonstrated that *Salmonella* targets Claudin-2 to facilitate bacterial invasion^18^, and epithelial cells with Claudin-2 knockdown have significantly less internalized *Salmonella* than control cells with normal Claudin-2 expression. Claudin-2 has also been identified as a target of Wnt/β-catenin signaling^19^, which is essential for intestinal development, and a recent study reported that Claudin-2 and -12 contributed to vitamin D-dependent calcium homeostasis^20^. Because of this latter report, and because Vitamin D and its receptor (VDR) are implicated in the pathogenesis of various intestinal illness, including IBD 21,22,23,24,25,26,27,28,29,30,31, we sought to determine how Claudin-2 is regulated by VDR signaling.

In the current study, we hypothesize that Claudin-2 is a direct target of the VDR. Using whole body VDR^-/-^ mice, intestinal epithelial VDR conditional knockout (VDR^ΔIEC^) mice, and cultured human intestinal epithelial cells; we perform a series of molecular and biochemical experiments *in vivo* and *in vitro* to investigate VDR regulation of Claudin-2 expression in enterocytes.

## Results

### Intestinal VDR deficiency in epithelial cells leads to reduction of Claudin-2 at the mRNA and protein levels

**Figure 1:**
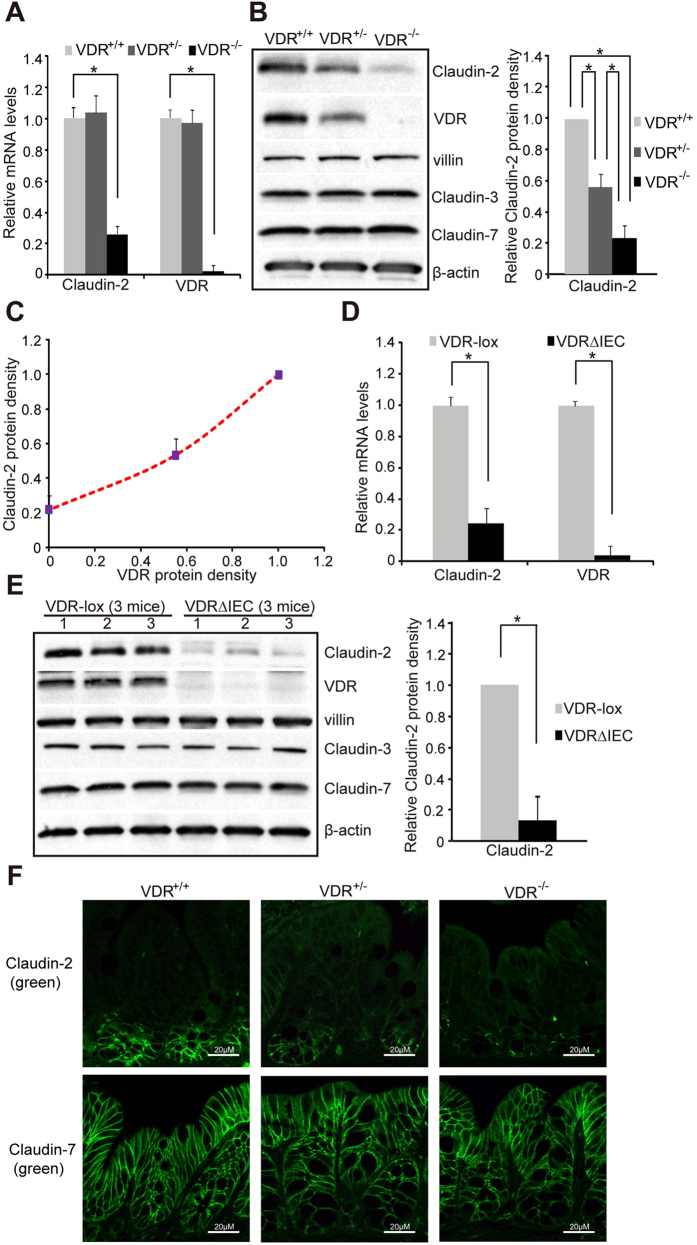
VDR status in intestinal epithelial cells leads to the change of Claudin-2 at both mRNA and protein levels *in vivo*. (**A**) Claudin-2 mRNA level and (**B**) Claudin-2 protein level in the intestinal epithelial cells of VDR^+/+^, VDR^+/-^, or VDR^-/-^ mice. (**C**) Claudin-2/VDR protein relative in the intestinal epithelial cells of VDR^+/+^, VDR^+/-^, or VDR^-/-^ mice. (**D**) Claudin-2 mRNA level and (**E**) Claudin-2 protein level in the intestinal epithelial cells of VDR KO (VDR^ΔIEC^) mice. Data are expressed as mean ± SD. *P < 0.05. n = 3 mice/group. (**F**) Location and quantification of Claudin-2 protein in colons of mice *in vivo.* Images for each protein shown represent three separate experiments. n = 3 mice/group.

In whole VDR^-/-^ mice, we detected significantly decreased mRNA levels of Claudin-2 in intestine (Fig. 1A), whereas other Claudins, such as Claudin-1,-4,-7,-10, and - 15, were not altered by the absence of VDR (data not shown). By immuno-blots, we further found that VDR^+/+^ mice had the highest protein level of Claudin-2 in intestine. VDR^+/-^ mice had intermediate levels of Claudin-2, whereas the VDR^-/-^ had the lowest levels of Claudin-2 protein (Fig. 1B). Therefore, VDR expression correlates with protein levels of Claudin-2 in colonic epithelial cells *in vivo* (Fig. 1C).

We further tested the specificity of intestinal VDR on expression levels of Claudin-2, in VDR^ΔIEC^ mice. We found that the mRNA levels of Claudin-2 were significantly lower in VDR^ΔIEC^ mice, compared to the VDR-lox mice (Fig. 1D). Claudin-2 protein was also significantly decreased in VDR^ΔIEC^, where no VDR protein was detected in VDR^ΔIEC^ colon by Western blot (Fig. 1E). As expected, Claudin-3 and Claudin-7 were unchanged. These data indicate that intestinal VDR specifically regulates the expression levels of Claudin-2.

Colonic Claudin-2 expression is uniquely restricted to the proliferative zone^10^,^11^,^18^. In Fig.1F, Claudin-2 staining (Green) was observed at the crypt base in VDR^+/+^ mice. The density of Claudin-2 fluorescence staining was weak in the VDR^+/-^ and VDR^-/-^ intestinal epithelial cells. Claudin-7 was very stable in these mice and showed unchanged distribution and density (Fig. 1F).

### Vitamin D_3_ treatment upregulates mRNA levels of Claudin-2

**Figure 2:**
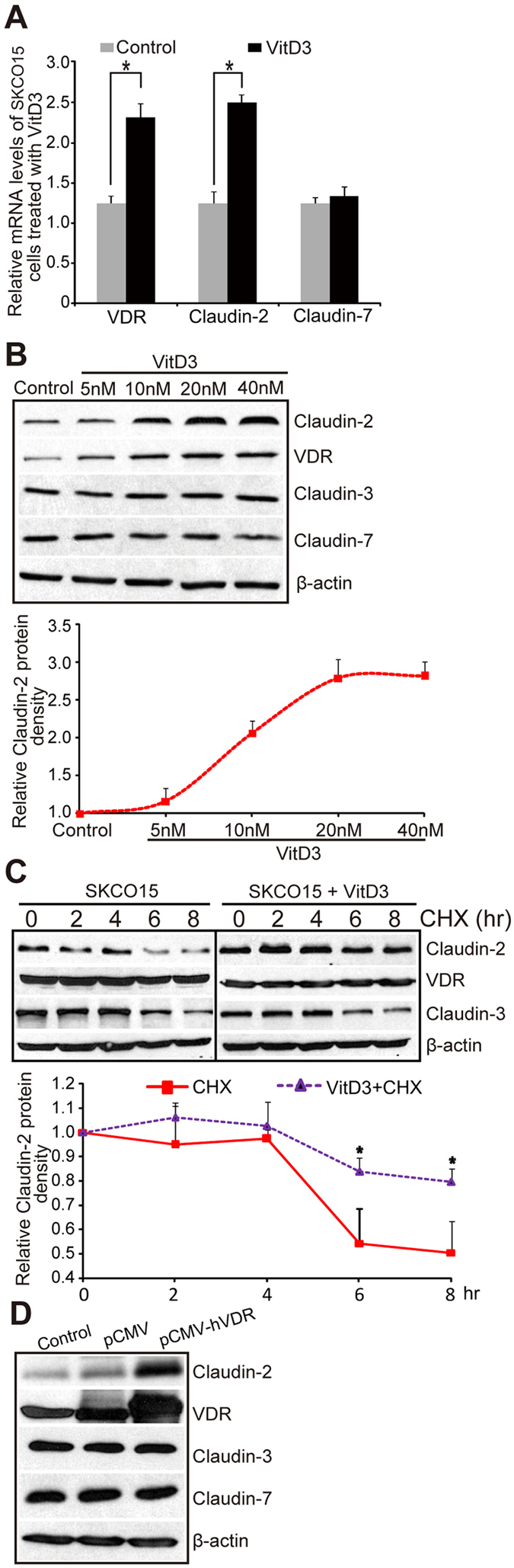
High levels VDR lead to increased Claudin-2 in human colonic epithelial SKCO15 cells *in vitro*. (**A**) Claudin-2 mRNA level increased post vitamin D_3_ treatment. SKCO15 cells were treated with vitamin D_3_ (20 nM) for 24 hours. Data are expressed as mean ± SD. *P < 0.05. n = 3 separate experiments. (**B**) Claudin-2 protein level and vitamin D_3_ dose-dependent curve. SKCO15 cells were treated with indicated vitamin D_3_ concentrations for 24 hours. Data are expressed as mean ± SD. *P < 0.05. n = 3 separate experiments. (**C**) Protein synthesis of Claudin-2 is high in vitamin D_3_-treated SKCO15 cells. Data are expressed as mean ± SD. *P < 0.05. n = 3 separate experiments. (**D**) Claudin-2 expression after SKCO15 cell transfection with human VDR in a pCMV-hVDR plasmid. Data are expressed as mean ± SD. *P < 0.05. n = 3 separate experiments.

For molecular mechanism studies *in vitro*, we used the human colonic epithelial SKCO15 cell line, which is widely used in studying TJs^32^,^33^. Vitamin D_3_ is known to increase VDR expression and activate VDR signaling. Claudin-2 mRNA was significantly elevated in SKCO15 cells treated with 1, 25 vitamin D_3_ (20 nM) for 24 hours, whereas Claudin-7 mRNA was not altered by vitamin D_3_ treatment (Fig. 2A). Moreover, protein levels of Claudin-2 were increased by vitamin D_3_ treatment in a dose-dependent manner (Fig. 2B). In contrast, the expression of Claudin-3 and 7 was unchanged in cells receiving vitamin D_3_ treatment. These data suggest that the Claudin-2 gene could be a direct transcriptional target of the VDR.

VDR is generally present in the cytosol or bound to DNA in an inactive state and requires activation by binding ligand^34^. Upon binding to vitamin D, VDR translocates to the nucleus and binds to vitamin D response elements (VDREs) in target genes and induces gene expression. A previous study showed that ongoing protein synthesis is not required for this process to occur^35^. We treated human SKCO15 cells with vitamin D_3_ (20 nM) in the presence or absence of cyclohexamide (CHX) to block protein synthesis. We chose to treat cells with vitamin D_3_ at 20 nM because our dose-response data in Fig. 2B indicated that 20 nm is a suitable concentration to induce Claudin-2 expression. CHX is an inhibitor of eukaryotic protein biosynthesis and is commonly used to determine protein half-life. Therefore, in the cells treated with CHX only, the expression of Claudin-2 was significantly decreased (Fig. 2C SKCO15). CHX+vitamin D_3_ treatment was able to stabilize the expression of Claudin-2 (Fig. 2C SKCO15+Vit.D3). Whereas vitamin D_3-_ induced Claudin-2 gene expression occurred in the absence of ongoing protein synthesis (presence of CHX), Vitamin D treatment did not induce Claudin-3 gene expression (Fig. 2C SKCO15+Vit.D3). These data further support the hypothesis that the Claudin-2 gene is a direct target of the VDR and not activated by secondary events, such as the synthesis of other transcription factors that are induced by VDR.

To study the effect of VDR overexpression on Claudin-2, we transiently transfected the human SKCO15 cells with a pCDNA-hVDR plasmid expressing human VDR. We found Claudin-2 expression increased after SKCO15 cells were transfected with pCMV-hVDR plasmids, whereas no change of Claudin-3 with VDR overexpression (Fig. 2D).

### VDR binds the Claudin-2 promoter *in vitro* and *in vivo*

**Figure 3:**
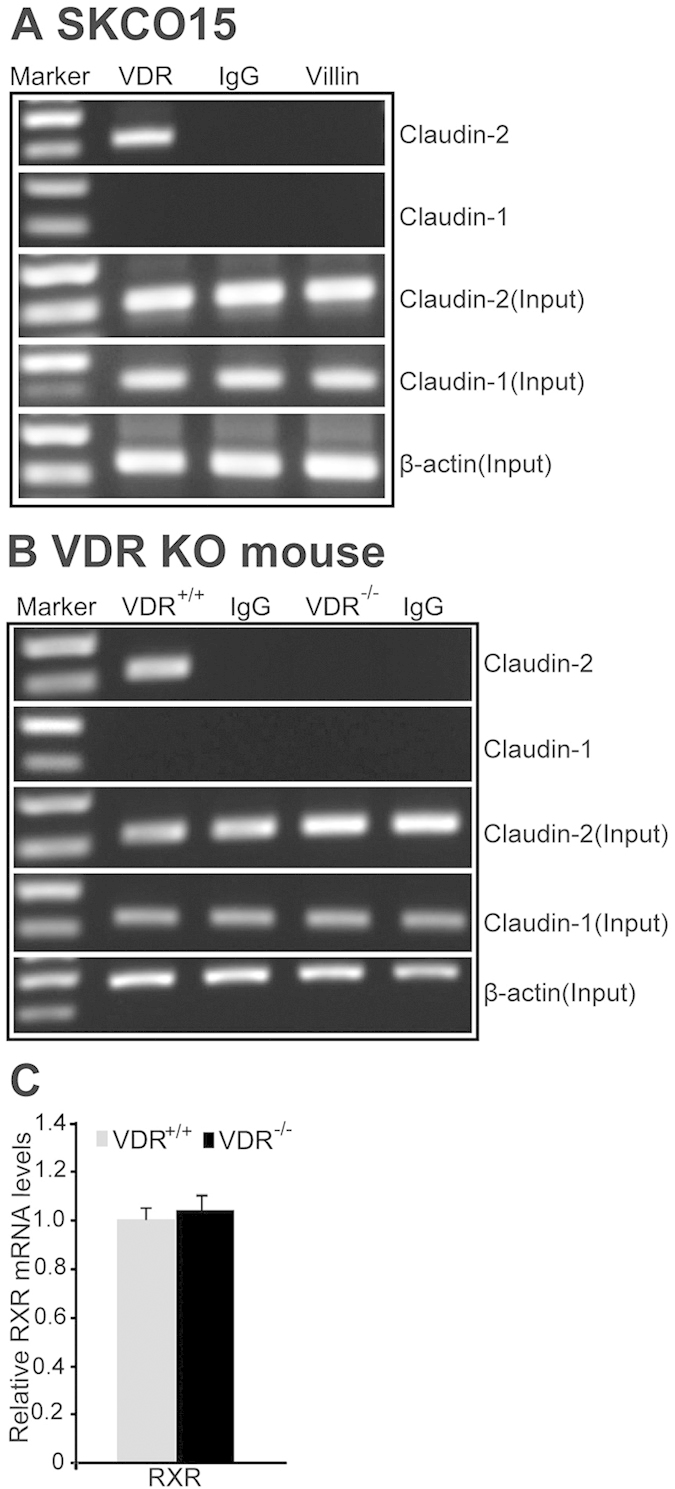
VDR binds to the Claudin-2 promoter. (**A**) CHIP-PCR amplification demonstrated binding of VDR to the promoter regions of human Claudin-2 in SKCO15 cells. PCR were performed including input-positive controls and IgG/villin-negative controls. n = 3 separate experiments. (**B**) ChIP-PCR assays of VDR binding to the Claudin-2 promoter in mouse colon. n = 3 separate experiments. (**C**) PCR analysis of RXR in VDR^+/+^ or VDR^-/-^ mice. Data are expressed as mean ± SD. *P < 0.05. n = 3 separate experiments.

VDR is a nuclear receptor that acts as a transcription factor to regulate expression of its target genes^21^,^36^. We reasoned that VDR may bind to DNA promoters of Claudin-2, thus changing mRNA expression of the Claudin-2 genes. VDR’s effect on promoters of Claudin-2 was analyzed by CHIP assay. We designed primers to the nonrepetitive region near the transcriptional start site that specifically amplifies the Claudin-2 promoter. For negative controls, chromatin was immunoprecipitated with IgG or villin. The samples were amplified by conventional PCR. We found that VDR bound to the Claudin-2 promoter *in vitro* (SKCO15 cells, Fig. 3A) *and in vivo* (mouse colon, Fig. 3B). The expression of the other Claudin members, such as Claudin-1, was also tested. We found that VDR did not bind to the Claudin-1 promoter, either *in vitro* or *in vivo* (Figs. 3A,B). VDR is known to interact with nuclear receptor RXR in regulating gene expression. However there was no significant change in mRNA level of RXR in the VDR^-/-^ intestine (Fig. 3C).

### Lacking VDR decreases Claudin-2 by abolishing VDR/promoter binding

**Figure 4:**
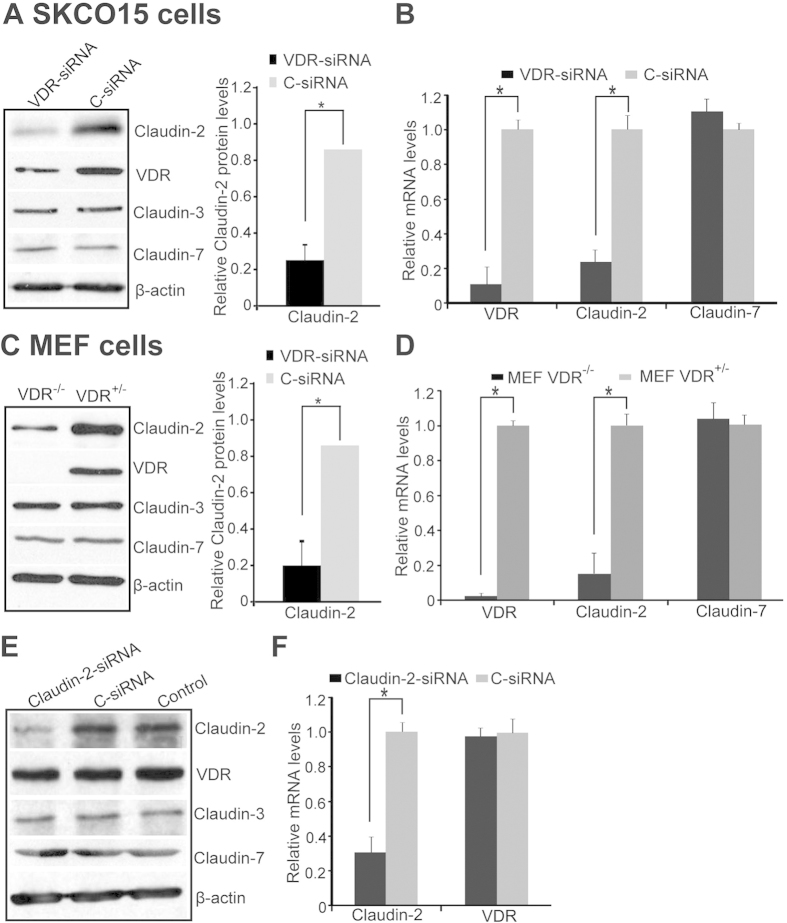
Lacking VDR decreases Claudin-2 protein and mRNA expression. (**A**) Claudin-2 protein and (**B**) mRNA were reduced by using siRNA against VDR. Data are expressed as mean ± SD. *P < 0.05. n = 3 separate experiments. (**C**) Claudin-2 protein and (**D**) mRNA were decreased in MEF VDR^+/-^/VDR^-/-^ cells. Data are expressed as mean ± SD. *P < 0.05. n = 3 separate experiments. (E) VDR protein and (**F**) mRNA did not change after Claudin-2 was knocked down with Claudin-2 siRNA for 72 hours in SKCO15 cells. Data are expressed as mean ± SD. *P < 0.05. n = 3 separate experiments.

We reasoned that less Claudin-2 was generated in cells lacking VDR if VDR binds to the promoter of Claudin-2. We further tested the effects of VDR in regulating mRNA and protein levels of Claudin-2 in human colonic epithelial SKCO15 cells with VDR-siRNA. We found that Claudin-2 mRNA and protein expression were reduced when VDR was knocked down by siRNA (Fig. 4A,B). To examined the effect of one allele of *VDR* gene on the expression of Claudin-2, we chose VDR^-/-^ and VDR^+/-^ mouse embryonic fibroblast (MEF) cells^37^. We found that one allele of the VDR gene in the VDR^+/-^ MEF cells was able to increase the expression of Claudin-2 protein (Fig. 4C). In contrast, Claudin-3 and -7 remained unchanged. At the transcriptional level, increased VDR mRNA was associated with elevated Claudin-2, but not Claudin-7 in VDR^+/-^ MEFs. Claudin-2 mRNA was significantly decreased in VDR^-/-^ MEF cells (Fig. 4D). This result suggests that VDR deletion affects Claudin-2 mRNA. Additionally, if we knocked down Claudin-2 by siRNA, there was no reduction of VDR at either the protein or mRNA level (Fig. 4E,F). These data suggest that Claudin-2 is downstream of VDR signaling.

### VDR-enhances Claudin-2 promoter activity in a Cdx1 binding site-dependent manner

**Figure 5:**
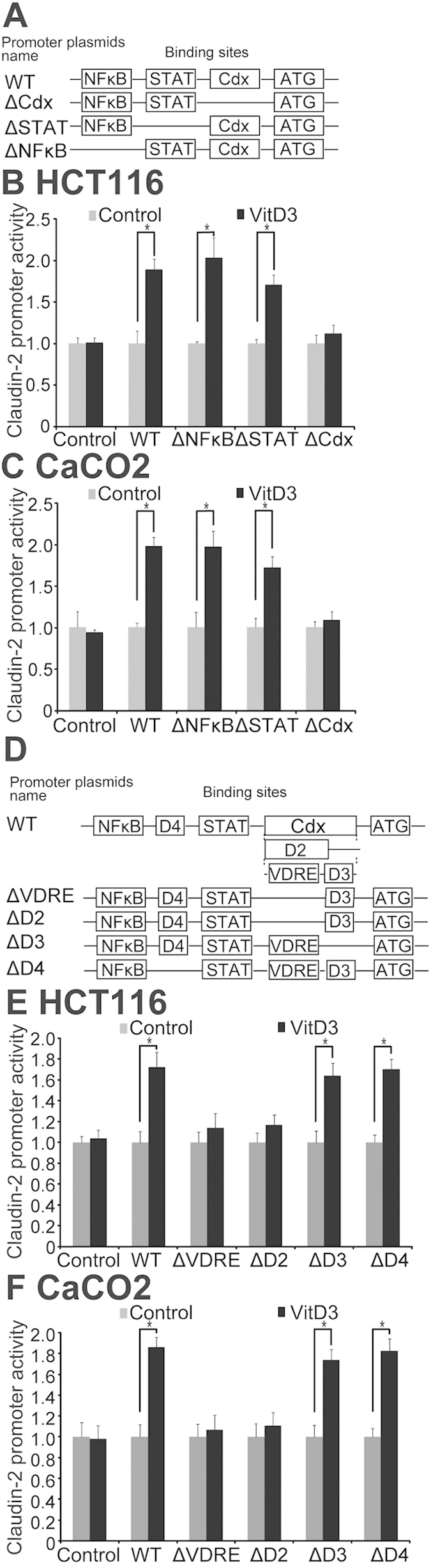
Identification of a functional VDRE sequence in the Claudin-2 promoter. (**A**) A schematic representation of transcriptional binding sites in the WT Claudin-2 promoter and deletion mutants. Plasmids include wild-type (WT), binding site deletions of NFκB (ΔNFκB), STAT (ΔSTAT), or Cdx1 (ΔCdx) in the Claudin-2 promoter. (**B**) WT or mutant Claudin-2 reporter plasmids were transfected in HCT116 and (**C**) CaCO2 cells. Luciferase activity was measured in the cell monolayers incubated in the absence or presence of vitamin D_3_ (20 nM) for 24 hours. Dual luciferase assays were performed and firefly luciferase activity was normalized to renilla luciferase activity. Data are expressed as mean ± SD. *P < 0.05. n = 3 separate experiments. (**D**) A schematic representation of VDRE deletion construct plasmids. Putative VDR-binding sites (containing AGATAACAAAGGTCA sequence) are designated as VDRE. Deletions of all VDRE binding sites (ΔVDRE), deletion of VDRE binding sites and adjacent bases (ΔD2), and non-VDRE deletion controls (ΔD3/ΔD4). (E) WT Claudin-2 reporter gene plasmids and the deletion mutant plasmids were transfected to HCT116 and (F) CaCO2 cells. Luciferase activity was measured in the cell monolayers incubated in the absence or presence of vitamin D_3_ (20 nM) for 24 hours. Data are expressed as mean ± SD. *P < 0.05. n = 3 separate experiments.

Cdx is a member of the caudal-related homeobox gene family^38^,^39^. Suzuki *et al.* reports that IL-6-induced Claudin-2 promoter activity requires Cdx binding sites^40^. To assess whether vitamin D3 could enhance Claudin-2 promoter activity through Cdx binding sites, we used an *in vitro* reporter Luciferase assay. A schematic drawing of transcriptional binding sites in the wild-type (WT) Claudin-2 promoter and its mutants is shown in Fig. 5A. Plasmids with WT or deletions of NFκB, STAT, or Cdx1 in the Claudin-2 promoter binding site (of ΔNFκB, ΔSTAT, or ΔCdx) were transfected into cells, respectively, and then treated with vitamin D_3_. Vitamin D_3_ enhanced WT-Claudin-2 promoter activity in both HCT116 and CaCO2 cells (Fig. 5B,C). Deletions of NFκB and STAT binding sites did not affect the Claudin-2 promoter activity. In contrast, deletions of Cdx1 binding sites clearly suppressed the promoter activity (Fig. 5B,C). These results demonstrate that vitamin D_3_-induced Claudin-2 expression requires Cdx1 binding sites in the Claudin-2 promoter sequence.

### Identification of a functional VDRE sequence in the Claudin-2 promoter

As Claudin-2 promoter activity is strongly elevated by exposure to 1, 25 vitamin D_3_, we predicted the existence of a VDRE in the Claudin-2 promoter. Our results from the reporter assay suggested that Cdx binding sites are involved in vitamin D_3_-mediated increases in Claudin-2 expression. Therefore, studies were conducted to investigate the Cdx binding site region. VDRE sequence is AGATAACAAAGGTCA^41^. A search of the Cdx region revealed a DR3-type, which binds preferentially to directly arrangements of two hexameric binding sites with three spacing nucleotides. PCR was used to construct deletions of all VDRE binding sites (ΔVDRE), deletion of VDRE binding sites and adjacent bases (ΔD2), and non-VDRE deletion controls (ΔD3/ΔD4). These fragments were separately subcloned into the pGL3-basic firefly luciferase reporter plasmid. A schematic drawing of the VDRE deletion and control mutants is shown in Fig. 5D. The Claudin-2 promoter VDRE deletion constructs were transfected into cells and were subsequently treated with vitamin D_3_ (20 nM). Deletions of VDRE (ΔVDRE and ΔD2) clearly lower the promoter activity of vitamin D_3_ in HCT116 (Fig. 5E) and CaCO2 cells (Fig. 5F). In contrast, non-VDRE deletion controls (ΔD3 and ΔD4) did not affect the Claudin-2 promoter activity induced by vitamin D_3_ (Fig. 5E&5F). Our results demonstrate that deletion of the VDRE sequence 5΄-AGATAACAAAGGTCA-3΄ in the Claudin-2 promoter region causes loss of its responsiveness to vitamin D_3_, and thus confirm that Claudin-2 is a direct target of vitamin D receptor signaling in intestinal epithelial cells.

## Discussion

The experimental focus of our current study was to investigate the molecular mechanisms whereby VDR may act as a transcriptional factor to regulate the expression of Claudin-2. First, we provide molecular biological evidence that the Claudin-2 gene is a direct target of the transcription factor VDR. A transcriptional reporter study demonstrated Claudin-2 up-regulation by over-expressed VDR. CHIP-PCR data demonstrated specific binding of VDR to the Claudin-2 promoter. VDR enhanced Claudin-2 promoter activity in a Cdx1 binding site-dependent manner. Next, we identified a functional VDRE sequence within in the Claudin-2 promoter. Knockout of VDR led to lower Claudin-2 at both mRNA and protein levels. Increased VDR by vitamin D_3_ pretreatment was associated with elevated Claudin-2 mRNA and protein levels. This study highlights an important and novel mechanism for VDR regulation of Claudin-2 critical to intestinal homeostasis.

Claudin-2 is a unique member of the Claudin family of transmembrane proteins as its expression is restricted to leaky epithelium *in vivo and* correlates with epithelial leakiness *in vitro*. VDR is a nuclear receptor that mediates most functions of vitamin D^50^,^51^,^52^. Our data showed that activation of the *CLDN2* gene occurred via a consensus VDRE in the promoter that is bound by VDR. VDR is expressed in a wide range of tissues. Therefore, potentially, Claudin-2 can be induced in various tissues. We know that multiple factors contribute to the upregulation of Claudin-2 at the transcriptional level^4^,^13^,^40^. TNF-α and IL-1beta contribute to elevated Claudin-2 *in vitro*^42^,^43^. IL-6 enhances claudin-2 promoter activity in a Cdx binding site-dependent manner^40^. VDR has multiple critical functions in regulating innate and adaptive immunity, intestinal homeostasis, host response to invasive pathogens and commensal bacteria, and tight junction structure^35^,^53^,^54^,^55^,^56^,^57^,^58^,^59^,^60^. TJ structure plays a critical role in intestinal barrier and inflammation^44^,^45^,^46^,^47^,^48^,^49^. Claudin-2 is enhanced in the inflamed gut of patients with IBD^12^,^17^. The pathobiological importance of the VDR regulation of Claudin-2 could be complex. Hence, further insight into the mechanisms responsible for VDR and barrier dysfunction in mucosal inflammation is needed, especially in *in vivo* systems and disease models.

In summary, for the first time, we identify *CLDN2* gene is a direct target of VDR. Our findings reveal a novel activity of VDR in regulation of TJs in primary cell structure and intestinal homeostasis. This study fills an existing gap by characterizing the precise molecular mechanism of VDR in regulating Claudin-2 and highlights the complex role of VDR in intestinal homeostasis^61^. It also brings up the possibility for restoration VDR-dependent functions and prevention of the intestinal barrier breakdown in patients with intestinal disorders.

## Materials and Methods

### Animals

VDR^+/+^, VDR^+/-^ and VDR^−/−^ mice on a C57BL6 background were obtained by breeding heterozygous VDR^+/−^ mice^62^. VDR flox mice were originally reported by Dr. Geert Carmeliet 63. VDR^ΔIEC^ mice were obtained by crossing the VDR flox mice with villin-cre mice (Jackson Laboratory, 004586, Bar Harbor, Maine, USA), as we previously reported^21^. Experiments were performed on 2–3 months old mice. All animal work was approved by the Rush University Committee on Animal Resources. Euthanasia method was sodium pentobarbital (100 mg per kg body weight) I.P. followed by cervical dislocation.

### Ethics statement

The methods in animal models were carried out accordance with the approved guidelines by the Rush University Committee on Animal Resources.

### Mouse colonic epithelial cells

Mouse colonic epithelial cells were collected by scraping the tissue from the colon of the mouse, including the proximal and distal regions^64^,^65^. The cells were sonicated in lysis buffer (1% Triton X-100, 150 mM NaCl, 10 mM Tris, pH 7.4, 1 mM EDTA, 1 mM EGTA, pH 8.0, 0.2 mM sodium ortho-vanadate, and protease inhibitor cocktail). The protein concentration was measured using the BioRad Reagent (BioRad, Hercules, CA, USA).

### Cell culture

Human epithelial CaCO2 and SKCO15 cells were maintained on transwell inserts (0.33 or 4.67 cm^2^, 0.4 mm pore. Costar, Cambridge, MA, USA) in DMEM supplemented with 10% fetal bovine serum, penicillin-streptomycin (Penicillin, 100 I.U./ml/Streptomycin, 100 μg/ml), and L-glutamine (4.5 g/L). Human colonic epithelial HCT116 cells, VDR^+/−^ and VDR^−/−^ MEF cells were cultured in DMEM medium supplemented with 10% (vol/vol) fetal bovine serum, as previously described^62^,^65^.

### Immunofluorescence

Colonic tissues were freshly isolated and embedded in paraffin wax after fixation with 10% neutral buffered formalin. Immunofluorescence was performed on paraffin-embedded sections (4 μm), after preparation of the slides as described previously^62^ followed by incubation for 1 hour in blocking solution (2% bovine serum albumin, 1% goat serum in HBSS) to reduce nonspecific background. The tissue samples were incubated overnight with primary antibodies at 4 °C. The following antibodies were used: anti-Claudin-2, anti-Claudin-7 (Invitrogen, Grand Island, NY, USA). Samples were then incubated with secondary antibodies (goat anti-mouse Alexa Fluor 488 or goat anti-rabbit Alexa Fluor 488, Molecular Probes, CA; 1:200) for 1 hour at room temperature. Tissues were mounted with SlowFade Antifade Kit (Life technologies, s2828, Grand Island, NY, USA), followed by a coverslip, and the edges were sealed to prevent drying. Specimens were examined with a Zeiss laser scanning microscope (LSM) 710 (Carl Zeiss Inc., Oberkochen, Germany).

### Analysis of claudins distribution

Fluorescence images were analyzed using image analysis software (LSM 710 META, version 4.2; Carl Zeiss Inc., Oberkochen, Germany). Each analysis was performed in triplicate from each tissue section on a total of 10 images per mouse sample (n = 5).

### Transient transfections

Transient transfections were performed with Lipofectamine 2000 (Invitrogen, San Diego, CA, USA) in accordance with the manufacturer’s instructions. Cells were seeded on 60 mm dishes overnight before transfection with DNA and were mixed with liposome reagent at a ratio of 1:1 before addition to cells. After a 24-hour transfection period, the proteins were extracted for western-blot analysis.

### Chromatin immunoprecipitation (ChIP) assays

The ChIP assays were performed essentially as described by the manufacturer (Upstate Inc., Chalottesville, VA, USA). Briefly, SKC015 cells or scraped VDR^+/+^/VDR^-/-^ colonic epithelial cells were treated with 1% formaldehyde for 10 min at 37 ^0^C. Cells were washed twice in ice-cold phosphate buffered saline containing protease inhibitor cocktail tablets (Roche, Nutley, NJ, USA). Cells were scraped into conical tubes, pelleted and lysed in SDS Lysis Buffer. The lysate was sonicated to shear DNA into fragments of 200–1000 bp (4 cycles of 10 s sonication, 10 s pausing, Branson Sonifier 250, Danbury, CT, USA). The chromatin samples were pre-cleared with salmon sperm DNA-bovine serum albumin-sepharose beads, then incubated overnight at 4 ^0^C with VDR antibody (Santa Cruz Biotechnology Inc., Dallas, Texas, USA). Immune complexes were precipitated with salmon sperm DNA-bovine serum albumin-sepharose beads. DNA was prepared by treatment with proteinase K, extraction with phenol and chloroform, and ethanol precipitation, and was subjected to PCR (Primers see supplement table 1).

### Western-blot analysis

Mouse epithelial cells were lysed in lysis buffer (1% Triton X-100, 150 mM NaCl, 10 mM Tris, pH 7.4, 1 mM EDTA, 1 mM EGTA, pH 8.0, 0.2 mM sodium ortho-vanadate, and protease inhibitor cocktail) and the protein concentration was measured. SKC015 and MEF cells were rinsed three times in ice-cold HBSS, lysed in protein loading buffer (50 mM Tris, pH 6.8, 100 mM dithiothreitol, 2% SDS, 0.1% bromophenol blue, and 10% glycerol) and sonicated (Branson Sonifier, 250). Equal amounts of protein were separated by SDS-polyacrylamide gel electrophoresis, transferred to nitrocellulose (162–0112, Bio-rad, Hercules, CA, USA), and immunoblotted with primary antibodies. The following antibodies were used: anti-Claudin-2, anti-Claudin-3, anti-Claudin-7 (Invitrogen, Grand Island, NY, USA), anti-VDR, anti-Villin (Santa Cruz Biotechnology Inc., Dallas, Texas, USA), or anti-β-actin (Sigma-Aldrich, St. Louis, MO, USA) and were visualized by ECL (Thermo Scientific, Rockford, IL, USA). Membranes that were probed with more than one antibody were stripped before reprobing.

### Transcriptional activation

After a 24 hour transfection period, the cells were lysed and luciferase activity was determined using the Dual Luciferase Reporter Assay System (Promega, Madison, WI, USA) with a TD-20/20 luminometer (Turner Designs, Sunnyvale, CA, USA). Firefly luciferase activity was normalized to *Renilla* luminescence activity and the activity was expressed as relative units.

### Identification of functional VDRE

PCR was used to construct deletion of entire VDRE binding sites (ΔVDRE), deletion of VDRE binding site with adjacent bases (ΔD2), and control (ΔD3/ΔD4). These fragments were separately subcloned into the firefly luciferase reporter plasmid pGL3-basic (Primers see supplement table 2). Deletions of different domains of the Claudin-2 promoter cloned into the in pGL3 vector, driving luciferase expression, were transfected into HCTC116/CaCO2 cells. Luciferase activity in cell lysates was assayed by the Dual Luciferase Reporter Assay System (Promega, Madison, WI, USA).

### Real-time quantitative PCR analysis

Total RNA was extracted from mouse epithelial cells or cultured cells using TRIzol reagent (Invitrogen, Grand Island, NY, USA). The RNA integrity was verified by electrophoresis. RNA reverse transcription was performed using the iScript cDNA synthesis kit (Bio-Rad, Hercules, CA, USA) according to the manufacturer’s protocol. The RT cDNA reaction products were subjected to quantitative real-time PCR using CTFX 96 Real-time system (Bio-Rad, Hercules, CA, USA) and SYBR green supermix (Bio-Rad, Hercules, CA, USA) according to the manufacturer’s protocol. All expression levels were normalized to β-actin levels of the same sample. Percent expression was calculated as the ratio of the normalized value of each sample to that of the corresponding untreated control cells. All real-time PCR reactions were performed in triplicate. Optimal primer sequences were designed using Primer-BLAST or were obtained from Primer Bank primer pairs listed in Supplement Table 3.

### Statistical Analysis

All of the data are expressed as means ± SD. All of the statistical tests were two-sided and P values of less than 0.05 were considered to be statistically significant. Differences between two samples were analyzed using Student’s t-test. The statistical analyses were performed using SAS version 9.2 (SAS Institute, Inc., Cary, NC).

## Additional Information

**How to cite this article**: Zhang, Y.-g. *et al*. Tight junction *CLDN2* gene is a direct target of the vitamin D receptor. *Sci. Rep.* **5**, 10642; doi: 10.1038/srep10642 (2015).

## Supplementary Material

Supplementary Information
