# Supplementary material for: Tight junction CLDN2 gene is a direct target of the vitamin D receptor
Source: Sci Rep. 2015 Jul 27;5:10642. doi: 10.1038/srep10642 (PMC4650691; doi:10.1038/srep10642)
Supplement: Supplementary Information [file srep10642-s1.pdf]

Tight junction *CLDN2* gene is a direct target of the vitamin D receptor

Yong-guo Zhang[1], Shaoping Wu[1], Rong Lu[1], David Zhou[2], Jingsong Zhou[3], Geert Carmeliet[4], Elaine Petrof[5], Erika C. Claud[6], Jun Sun[1,2]

1. Department of Biochemistry, Rush University, Chicago, Illinois, USA
2. Department of Pathology, University of Rochester, Rochester, New York, USA
3. Department of Physiology, Kansas City University of Medicine and Bioscience, Kansas City, Missouri, USA
4. Lab of Experimental Medicine and Endocrinology, Katholieke Universiteit Leuven, Leuven, Belgium
5. Department of Medicine, GI Diseases Research Unit and Division of Infectious Diseases, Queen's University, Ontario, Canada
6. Departments of Pediatrics and Medicine, The University of Chicago Medical Center, Chicago, Illinois, USA

Supplementary Figure Legends

Supplementary Figure 1. Sequence analysis of the 5´-flanking region of the human Claudin-2 gene. Potential binding sites are underlined.

```
   1 GAATCTTGGC AACACCGAGG GCTCCTTGAA CACGGCAAAA TCTTATATGG CTCTGAGATT

  61 CCAAAGCATT GACTCAGATA CCTGCCTCAT GCAAAGCCCT ATATTCTAGA GCAGTTTCCC

 121 TTTCCTCTGT GGCAGACTCT TGTCCCCCCT AACAGATGGC CCAGGGAATT TCAGGGCCCC
                                                     NFκB
 181 CTCTCAGTCC TGGAACCCTT GTTCCAGAGT GCTCCCTCAT CATCCAAGAG GCTGATGATG

 241 GGAGCATCTA TTAGGAGACT GGACAGGAAA TGTCTGGGCA TGTTATACAT GCAGGAGGCC

 301 TTAGACTAGG CTGCAGAGGG GGATTTGGGC ATGGCTGGGA GGATCTGAAC TCTCAGAGTA

 361 TGGACAGAAG GCTTTGCTGC CCACCCCCAT CTACCCTGGA GTAGATTTTC ACCATGGGCA

 421 GAATGATCCA GGGCTAGGCC ACTACTCTCT AGGCCCCTGG AGATTCAAGA GGCCTCTAAC

 481 AAACTGGAGT CCAAGACTAC ATTCTAGGAT CTGTTCCTCC TGATGTAGTC TGCAGTTTGG

 541 CCTCAGTCTG CAATTGAGGG GCCCTATGGC ACTGTTGCTT GGCAATGTAT TAAACAGCAG

 601 GCCTTGGAGA CTAGCACTTG AGTTAACACA GCCACCACAA CCACCACTGC CATCATCACC

 661 TTCCCGGAAA GCAGCCACCT GTCTGGCTCC TGGCTTTGTC CAGCTGCCAA CCTAAGGCAT
        STAT
 721 GTGCCTACGC AGGAGGCGAT GACATTTTGG CTCCACGTTC AAAGTTGTTT TTTTTTTCCT

 781 TTCTCATGTG TTATTTCTAA AGATAACAAA GGTCAAAAGG CATCCAGCGT TTTCTGGTTT
                 Cdx-A        VDRE                               Cdx-B
 841 CTCATAAGCT TCTGGTCAAT ATTTAATCTG GTTTATGGAT TTTTTTTAGG TCTTCTAGAT
                                   Cdx-C          Cdx-D
 901 GCCTTCTTGA GGCTGCTTGT GGCCACCCAC AGACACTTGT AAGGAGGAGA GAAGTCAGCC
                             Start of Transcription
 961 TGGCAGAGAG ACTCTGAAAT GAGGGATTAG AGGTGTTCAA GGAGCAAGAG CTTCAGCCTG

1021 AAGACAAGGG AGCAGTCCCT GAAGACGCTT CTACTGAGAG GTCTGCCATG
                                                   Start of Translation
```

Supplement Table1: CHIP Primers

Primers name	Sequence
CMC2F	5´-TGCCAGCCTAAAACATGTGC-3´
CMC2R	5´-AGATTAAATATTGACCAGAAG-3´
CMC1F	5´-GGGATGCCTGATTCTCTTCA-3´
CMC1R	5´-TGGAAGTGAGAAGTGGCAGA-3´
CMactinF	5´-TCGATATCCACGTGACATCCA-3´
CMactinR	5´-GCAGCATTTTTTTACCCCCTC-3´
CHC2F	5´-GTCCAGCTGCCAACCTAAGGCA-3´
CHC2R	5´-CAGATTAAATATTGACCAGAAG-3´
CHC1F	5´-TGGAGGCATCGCTTGTTTTC-3´
CHC1R	5´-TAGCTGTCCTCTCTGCGTCT-3´
CHactinF	5´-GAGCACAGAGCCTCGCCTTT-3´
CHactinR	5´-AGACAAAGACCCCGCCGGTT-3´

Supplement Table 2: VDRE construct PCR Primers

**Primers name**	**Sequence**
ΔVDREF	5´-ATTACGCGTGGGTCTTGGCAACACCGA-3´
ΔD1F	5´-CCAGAAAACGCTGGATGCCGAAATAACACATGA-3´
ΔD1R	5´-CTCATGTGTTATTTCGGCATCCAGCGTTTTCTGG-3´
ΔD2F	5´-GACCAGAAGCTTATGAGAAACAACACATGAGAAAGG-3´
ΔD2R	5´-CCTTTCTCATGTGTTGTTTCTCATAAGCTTCTGGTC-3´
ΔD3F	5´-GAAGGCATCTAGAAGACGATTAAATATTGACCAG-3´
ΔD3R	5´-CTGGTCAATATTTAATCGTCTTCTAGATGCCTTC-3´
ΔD4F	5´-CATCTACCCTGGAGTAGATCCAGGGCTAGGCCACTA-3´
ΔD4R	5´-TAGTGGCCTAGCCCTGGATCTACTCCAGGGTAGATG-3´
ΔVDRER	5´-GCAGATCTGGCAGACCTCTCAGTAGAAG-3´

Supplement Table 3: Real-time PCR Primers

Primers name	Sequence
mβ-actinF	5´-TGTTACCAACTGGGACGACA-3´
mβ-actinR	5´-CTGGGTCATCTTTTCACGGT-3´
mVDRF	5´-GAATGTGCCTCGGATCTGTGG-3´
mVDRR	5´-ATGCGGCAATCTCCATTGAAG-3´
mClaudin2F	5´-GCAAACAGGCTCCGAAGATACT-3´
mClaudin2R	5´-GAGATGATGCCCAAGTACAGAG-3´
mClaudin7F	5´-GCGACAACATCATCACAGCC-3´
mClaudin7R	5´-CCTTGGAGGAATTGGACTTGG-3´
hβ-actinF	5´-AGAGCAAGAGAGGCATCCTC-3´
hβ-actinR	5´-CTCAAACATGATCTGGGTCA-3´
hVDRF	5´-GGACTGCCGCATCACCAA-3´
hVDRR	5´-TCATCTCCCGCTTCCTCT-3´
hClaudin2F	5´-ACCTGCTACCGCCACTCTGT-3´
hClaudin2R	5´-CTCCCTGGCCTGCATTATCTC-3´
hClaudin7F	5´-CATCGTGGCAGGTCTTGCC-3´
hClaudin7R	5´-GATGGCAGGGCCAAACTCATAC-3´

Supplement Table 4: Binding sites sequences for VDRE deletion constructs

Binding sites name	Sequence
VDRE	AGATAACAAAGGTCA
D2	TAAAGATAACAAAGGTCAAAA
D3	TGGTTTATGGATTTTTTTTAG
D4	CCTGGAGTAGATTTTCACC
